# Regulation of myotube formation by the actin-binding factor drebrin

**DOI:** 10.1186/2044-5040-1-36

**Published:** 2011-12-08

**Authors:** Annalisa Mancini, Dario Sirabella, Weijia Zhang, Hiroyuki Yamazaki, Tomoaki Shirao, Robert S Krauss

**Affiliations:** 1 Department of Developmental and Regenerative Biology, Mount Sinai School of Medicine, One Gustave L. Levy Place, New York, NY 10029, USA; 2 Department of Medicine, Mount Sinai School of Medicine, One Gustave L. Levy Place, New York, NY 10029, USA; 3 Department of Neurobiology and Behavior, Gunma University Graduate School of Medicine, 3-39-22, Showa-machi, Maebashi, Gunma 371-8511, Japan

**Keywords:** myoblast, cell differentiation, drebrin, myotube, actin

## Abstract

**Background:**

Myogenic differentiation involves cell-cycle arrest, activation of the muscle-specific transcriptome, and elongation, alignment and fusion of myoblasts into multinucleated myotubes. This process is controlled by promyogenic transcription factors and regulated by signaling pathways in response to extracellular cues. The p38 mitogen-activated protein kinase (p38 MAPK) pathway promotes the activity of several such transcription factors, including MyoD and MEF2, thereby controlling the muscle-specific transcription program. However, few p38-regulated genes that play a role in the regulation of myogenesis have been identified.

**Methods:**

RNA interference (RNAi), chemical inhibition and immunofluorescence approaches were used to assess the role of drebrin in differentiation of primary mouse myoblasts and C2C12 cells.

**Results:**

In a search for p38-regulated genes that promote myogenic differentiation, we identified *Dbn1*, which encodes the actin-binding protein drebrin. Drebrin is an F-actin side-binding protein that remodels actin to facilitate the change of filopodia into dendritic spines during synaptogenesis in developing neurons. *Dbn1 *mRNA and protein are induced during differentiation of primary mouse and C2C12 myoblasts, and induction is substantially reduced by the p38 MAPK inhibitor SB203580. Primary myoblasts and C2C12 cells depleted of drebrin by RNAi display reduced levels of myogenin and myosin heavy chain and form multinucleated myotubes very inefficiently. Treatment of myoblasts with BTP2, a small-molecule inhibitor of drebrin, produces a phenotype similar to that produced by knockdown of drebrin, and the inhibitory effects of BTP2 are rescued by expression of a mutant form of drebrin that is unable to bind BTP2. Drebrin in myoblasts is enriched in cellular projections and cell cortices and at regions of cell-cell contact, all sites where F-actin, too, was concentrated.

**Conclusions:**

Our findings reveal that *Dbn1 *expression is a target of p38 MAPK signaling during myogenesis and that drebrin promotes myoblast differentiation.

## Background

Myoblast differentiation is a multistep process that involves withdrawal from the cell cycle, acquisition of a cell type-specific transcriptional program and morphological changes that include elongation, alignment and fusion of myoblasts to form myofibers [1-4]. Whereas transcriptional regulation is at the core of myogenesis, the formation and growth of myofibers is also controlled by a variety of signaling ligands and their receptors, including insulin-like growth factor 1, fibroblast growth factors (FGFs), Wnts, transforming growth factor β superfamily members and others [1-3,5]. Furthermore, the activity of MyoD and other promyogenic transcription factors is tightly controlled at the posttranslational level by signal transduction pathways, including phosphatidylinositol 3-kinase/Akt, integrin/focal adhesion kinase (FAK) and calcium/calcineurin [4,6-9]. Among the signaling pathways that promote myogenesis, the p38α/β MAPK (p38α/β mitogen-activated protein kinase) pathway plays a prominent role. There is a persistent rise in p38α/β (hereafter simply "p38") activity during myoblast differentiation, and inhibition of p38 expression or activity blocks induction of select muscle-specific genes and myogenic differentiation [8,10-13]. p38 phosphorylates substrates that drive muscle-specific gene expression at several levels, including MyoD dimerization with E proteins, Mef2 transcriptional activity, chromatin remodeling at muscle-specific genes and stability of myogenic mRNAs [11,13-18]. Despite progress in understanding these proximal targets of p38's promyogenic actions, few p38-regulated genes that play a role in the regulation of myogenesis have been identified.

In addition to the acquisition of a muscle-specific transcriptional program, the changes in myoblast morphology that occur during differentiation indicate that dramatic alterations in the F-actin cytoskeleton are required for the formation of myofibers. Consistent with this notion, lamellipodia and filopodia, cellular structures that require actin remodeling, form dynamically during myoblast differentiation *in vitro *[19-21]. Furthermore, disruption of the F-actin cytoskeleton by chemical or other means inhibits various aspects of myoblast differentiation, including myoblast fusion [21-24]. At least some aspects of the cytoskeletal rearrangement and morphological changes that occur during differentiation are likely to be mediated by transcriptional induction of regulators of these processes, as expression of MyoD in fibroblasts induces not only expression of muscle-specific genes but also elongation and fusion into multinucleated myotubes [4].

Drebrin is a ubiquitously expressed F-actin side-binding protein that is highly abundant in the brain [25,26]. Drebrin contains an actin-depolymerizing factor II/cofilin-like domain, an actin-binding domain and two Homer-binding domains [27], and it remodels actin to facilitate the maturation of filopodia into dendritic spines during synaptogenesis in developing neurons (for reviews, see [27,28]). It is localized in lamellipodia and filopodia, at sites of cell-cell contact and in adhesion plaques [27-32]. Furthermore, drebrin associates with several proteins that promote myoblast differentiation and/or fusion, including the microtubule plus-tip binding protein EB3 [33,34]; the scaffold protein Homer [35,36] and, via Homer, the small GTPase Cdc42 [35,37,38]; and the chemokine receptor CXCR4 [39,40]. We report herein that drebrin expression is induced during differentiation of primary and C2C12 myoblasts in a p38 MAPK-dependent manner. Furthermore, depletion of drebrin by RNA interference (RNAi) or inhibition of its function with a small-molecule antagonist diminished expression of muscle-specific genes and myotube formation. Drebrin is therefore an actin-regulating factor induced during myogenic differentiation that promotes the differentiation process.

## Methods

### Cell culture

Primary myoblasts were isolated from wild-type mice at postnatal day 13 by the method of Rando and Blau [41] and maintained as previously described [42]. Differentiation was induced by switching cultures from Ham's F-10 medium with 20% fetal bovine serum (FBS)/2.5 ng/ml basic FGF (R&D Systems, Minneapolis, MN, USA)/4% penicillin-streptomycin (growth medium (GM)) to DMEM with 5% horse serum (differentiation medium (DM)). C2C12 myoblasts were maintained in DMEM containing 15% FBS supplemented with 1% penicillin/streptomycin and L-glutamine (GM). Differentiation was induced by transferring to DMEM supplemented with 2% horse serum (DM). Where indicated, 5 μM SB203580 (Sigma-Aldrich, St Louis, MO, USA) or 5 μM BTP2 (Calbiochem, La Jolla, CA, USA) were added to cultures and replenished every 12 hours.

### Expression of drebrin

Plasmids encoding murine GFP-tagged drebrin E were generated by subcloning cDNA encoding either wild-type murine *Dbn1 *E or the K270M, K271M mutant into the pEGFP-C1 vector (Clontech Laboratories, Inc, Mountain View, CA, USA). Plasmids were transfected into C2C12 cells using Lipofectamine 2000 reagent (Invitrogen/Life Technologies, Carlsbad, CA, USA) according to the manufacturer's instructions.

### RNAi

For small hairpin RNA studies, four sequences against murine *Dbn1 *were initially chosen. The oligonucleotides were cloned into the pSUPER.puro vector (Oligoengine, Seattle, WA, USA) and transfected into C2C12 cells with Lipofectamine 2000 reagent. Puromycin-resistant cells were selected, pooled and examined by Western blot analysis for drebrin expression. The most effective sequences were chosen for further studies and corresponded to nucleotides 5' GCCACTTCGAGAACCAGAAAG 3' (si*Dbn1*-1) and 5' AGGAAGAGCCATGTGCAAAGGT 3' (si*Dbn1*-3) (NM_019813.3).

Twenty-four hours after selection, cultures were seeded onto growth factor-reduced Matrigel (R&D Systems)-coated dishes at a density of 150,000 cells/ml. Twenty-four hours later, cultures were switched to DM. At various time points later, cultures were fixed with 2% paraformaldehyde (PFA) and immunostained for myosin heavy chain (MHC) and drebrin expression. Sister cultures were lysed, and 30 μg of cleared cell extracts were examined by Western blot analysis as previously described [43]. Drebrin expression was also knocked down by transfection of C2C12 cells or primary myoblasts with 200 nM drebrin-specific RNAi (4390771; Ambion/Life Technologies) or with 200 nM control nonsilencing RNAi Red (465318; Invitrogen/Life Technologies). RNAs were transfected with StemPro LipoMax reagent (Invitrogen/Life Technologies) according to the manufacturer's instructions. Thirty-six hours after transfection, cells were either harvested to assess drebrin expression by Western blot analysis or analyzed for differentiation potential tested by transfer to DM.

### Protein analysis

Western blot analyses were performed as described in Kang *et al. *[43]. Briefly, cells were lysed in extraction buffer (50 mM Tris·HCl, pH 7.4, 50 mM NaF, 5 mM sodium pyrophosphate, 1 mM sodium orthovanadate, 1 mM ethylenediaminetetraacetic acid, 1% Triton X-100) supplemented with protease and phosphatase inhibitor cocktail (Sigma-Aldrich). Total proteins were resolved on SDS-polyacrylamide gels, transferred onto Immobilon polyvinylidene fluoride membranes (Millipore, Billerica, MA, USA) and probed with specific antibodies. Primary antibodies used were anti-drebrin (Abcam, Cambridge, MA, USA), anti-tubulin (Santa Cruz Biotechnology, Santa Cruz, CA, USA), anti-myogenin (F5D; Santa Cruz Biotechnology) and anti-MHC (MF20; Developmental Studies Hybridoma Bank, Department of Biology, University of Iowa, Iowa City, IA, USA). Membranes were reprobed with the appropriate horseradish peroxidase-conjugated secondary antibody (The Jackson Laboratory, Bar Harbor, ME, USA), and specific bands were visualized with an enhanced chemiluminescence detection system (Roche Applied Science, Indianapolis, IN, USA)

### RNA analysis

RNA was isolated using the RNeasy Mini Kit (QIAGEN, Valencia, CA, USA). One microgram of total RNA was reverse-transcribed using the First-Strand cDNA Synthesis Kit (Invitrogen/Life Technologies). One-tenth of the cDNA was applied for real-time PCR using QuantiFast SYBR Green RT-PCR Kit (QIAGEN) and analyzed on a Bio-Rad Q5 cycler (Bio-Rad Laboratories, Hercules, CA, USA).

*Dbn1*, *Myh3*, *Myog *and *Gapdh *mRNA expression were quantified with the following primers. *Dbn1 *forward: 5' AGGCCAAGAAGGAGGAAGAG 3'; *Dbn1 *reverse: 5' TTCCTCCTGTGCTCCTCAAT 3'; *Myh3 *forward: 5' CAGAAATGGAGACACGGATCAGA 3'; *Myh3 *reverse: 5' AGAGGTGAAGTCACGGGTCTTTGCC 3'; *Myog *forward: 5'GGGCCCCTGGAAGAAAAG 3'; *Myog *reverse: 5'AGGAGGCGCTGTGGGAGT 3'; *Gapdh *forward: 5' TGCACCACCAACTGCTTA 3'; *Gapdh *reverse: 5' GATGCAGGGATGATGTTC 3'.

### Immunostaining and microscopy

Cells were grown on either Matrigel- or collagen-coated dishes as indicated, fixed with 2% PFA for 10 minutes, permeabilized with 0.1% Triton X-100 for 5 minutes, blocked with PBS containing 5% goat serum for 1 hour and incubated overnight with the following primary antibodies: anti-MHC (clone MF-20; Developmental Studies Hybridoma Bank), anti-drebrin (rabbit; Abnova, Walnut, CA, USA) and Alexa Fluor 568 phalloidin (Invitrogen/Life Technologies). After being washed three times with PBS, cells were incubated with the appropriate secondary antibody conjugated with Alexa Fluor 488 or 568 (Invitrogen/Life Technologies) for 1 hour, counterstained with 4',6-diamidino-2-phenylindole (DAPI) and then visualized using a Nikon Eclipse TS100 inverted microscope (Nikon Instruments, Melville, NY, USA). Images were acquired using a ProgRes digital microscope camera system and software (Jenoptik AG, Jena, Germany).

### Cell proliferation and cell death assays

Cell proliferation was assessed by bromodeoxyuridine (BrdU) incorporation as described by Kang *et al. *[44]. Briefly, dividing cells were incubated in 20 mM BrdU for 2 hours. The medium was aspirated, and cells were immediately fixed for 30 minutes at -20°C with ice-cold 70% ethanol/30% glycine at pH 2.0. After being washed with PBS, cells were incubated with mouse anti-BrdU (Chemicon International/Millipore, Temecula, CA, USA) and anti-mouse Alexa Fluor 568 (Invitrogen/Life Technologies). Cells were counterstained with DAPI and visualized by the standard immunofluorescence protocol. Apoptosis was analyzed with the In Situ Cell Death Detection Kit (Roche Applied Science) according to the manufacturer's instructions.

## Results

### *Dbn1 *is induced during myogenic differentiation

**Figure 1:**
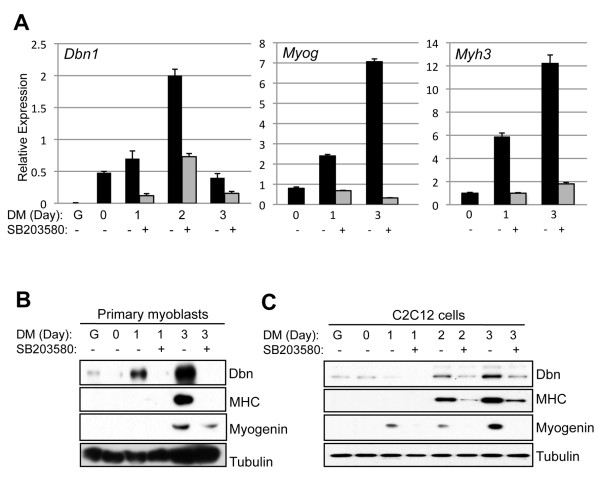
***Dbn1 *is upregulated during myoblast differentiation in an SB203580-sensitive manner**. **(A) **Primary mouse myoblasts proliferating in growth medium (G) or transferred to differentiation medium for the indicated times in the presence (+) or absence (-) of 5 μM SB203580 were analyzed by quantitative RT-PCR for expression *Dbn1*, *Myog *(encoding myogenin) and *Myh3 *(encoding myosin heavy chain 3; MHC3). **(B) **Cultures parallel to those in part **(A) **were examined by Western blot analysis for drebrin, myogenin and MHC production. Tubulin was used as a loading control. **(C) **C2C12 myoblasts were analyzed similarly to the primary myoblasts shown in part **(B)**.

In an effort to identify genes regulated by p38 signaling during myoblast differentiation, microarray analyses were performed on C2C12 cells cultured at low density in GM or DM at various time points, plus or minus the p38 inhibitor SB203580. Details of the screen will be published elsewhere. Herein we describe studies of one such gene identified in this screen, *Dbn1*, which encodes drebrin. Quantitative RT-PCR (qRT-PCR) analysis revealed that *Dbn1 *is expressed at very low levels in proliferating primary myoblasts in GM, is expressed at higher levels at the time of shift to DM (day 0, when cells have reached near-confluence) and increases in abundance over the next 2 days in DM (Figure 1A). Analysis of *Myog *and *Myh3 *expression served as a positive control for differentiation. *Dbn1 *expression during myoblast differentiation was biphasic, as mRNA levels decreased again after 3 days in DM. Parallel cultures treated with SB203580 displayed strongly dampened induction of *Dbn1*, *Myog *and *Myh3 *expression over this time course (Figure 1A). There are two alternatively spliced isoforms of drebrin: A, which contains a brain-specific exon, and E, which lacks this exon and is expressed broadly [27,28]. qRT-PCR with pan-drebrin or drebrin A-specific primers revealed, as expected, that drebrin E is the isoform expressed in myoblasts (data not shown). Drebrin protein levels in differentiating cultures of primary myoblasts and C2C12 cells were regulated similarly to *Dbn1 *mRNA in that they were induced during differentiation in an SB203580-sensitive manner, except that drebrin protein levels did not decrease after 3 days in DM; rather, they were still at their peak at this time point (Figures 1B and 1C). These results suggest that drebrin protein may be more stable than the mRNA that encodes it. In some Western blots, two distinct drebrin bands were resolved (e.g., Figure 1C). Treatment of cell lysates with alkaline phosphatase led to loss of the more slowly migrating band but had no effect on the mobility of the lower band, indicating that the upper band, as reported previously [45], is a phosphorylated form of drebrin (data not shown). The ratios of the two bands did not change as drebrin protein levels rose during differentiation, suggesting that phosphorylation is not a differentiation-dependent or regulated event.

### RNAi-mediated depletion of drebrin inhibits myoblast differentiation and myotube formation

**Figure 2:**
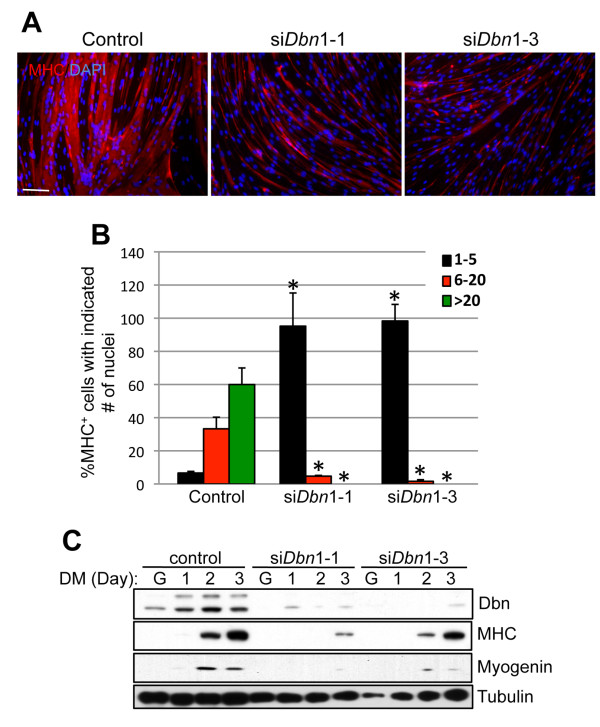
**RNA interference (RNAi)-mediated depletion of drebrin reduces C2C12 cell differentiation**. **(A) **C2C12 cells were transfected with either pSUPER (control) or pSUPER containing RNAi sequences targeted against *Dbn1 *(si*Dbn1*-1, si*Dbn1*-3). After 3 days in differentiation medium (DM), cultures were fixed and double-stained for myosin heavy chain (red) and 4',6-diamidino-2-phenylindole (blue). Bar: 0.1 mm. **(B) **Quantification of myotube formation. Values shown are means ± SD of triplicate determinations. *P < 0.05. **(C) **Western blot analysis of C2C12 cells expressing the indicated control or *Dbn1 *siRNA vectors cultured in growth medium (G) or DM for the indicated times.

**Figure 3:**
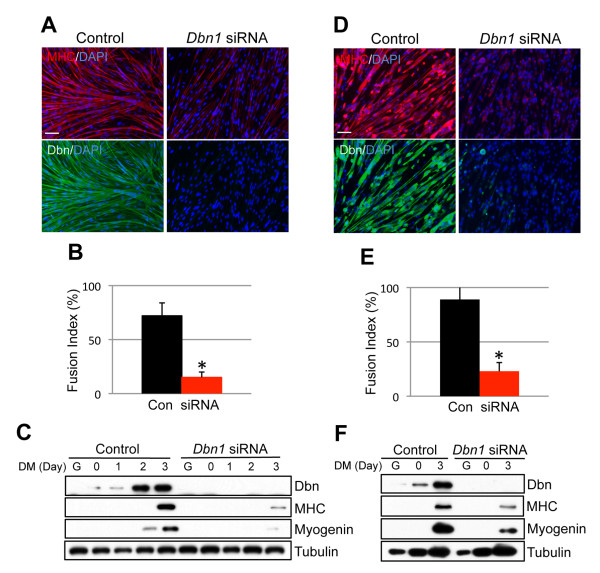
**siRNA-mediated depletion of drebrin inhibits differentiation of primary myoblasts**. **(A) **C2C12 cells were transfected with nonsilencing (control) or *Dbn1*-specific siRNA. After 3 days in differentiation medium, cultures were fixed and triple-stained for myosin heavy chain (MHC; red), drebrin (green) and 4',6-diamidino-2-phenylindole (blue). Bar: 20 μM. **(B) **Quantification of fusion index in cultures from part **(A)**. Con, control siRNA. Values shown are means ± SD of triplicate determinations. *P < 0.01. **(C) **Cultures parallel to those in part **(A) **were examined by Western blot analysis for drebrin, myogenin and MHC production. Tubulin was used as a loading control. **(D) **through **(F) **are the same as parts **(A) **through **(C)**, except primary myoblasts were used.

To explore a role for drebrin in myogenesis, an RNAi approach was taken. Two independent *Dbn1 *sequences were placed into the pSuper.puro vector and were individually and stably expressed in C2C12 cells. A vector harboring an irrelevant sequence was used as a control. Induction of drebrin was substantially dampened during differentiation of C2C12 cells that expressed either siRNA sequence, relative to control transfectants (Figure 2C). Depletion of drebrin with either sequence resulted in a similar strong reduction in the formation of multinucleated myotubes. Whereas control cells formed large myotubes, the majority of which had more than 20 nuclei, drebrin-depleted cells formed thin myotubes with fewer than five nuclei (Figures 2A and 2B). Production of the differentiation markers myogenin and MHC was somewhat variably affected in these cell lines, but each accumulated lower amounts of these muscle-specific proteins than did control transfectants during 3 days in DM (Figure 2C). To explore this further, we directly transfected siRNAs corresponding to a third *Dbn1 *sequence into both C2C12 cells and primary myoblasts. In both cell types, drebrin protein was barely detectable after knockdown (Figures 3A, C, D and 3F). Expression of myogenin and MHC in these cultures was observed by Western blotting and immunofluorescence, but was diminished relative to control cultures (Figures 3A, C, D and 3F). Formation of multinucleated myotubes was strongly inhibited in both C2C12 and primary myoblasts by anti-*Dbn1 *siRNA (Figures 3A, B, D and 3E). Taken together, these results indicate that drebrin promotes myoblast differentiation, as assessed by both biochemical and morphological criteria.

**Figure 4:**
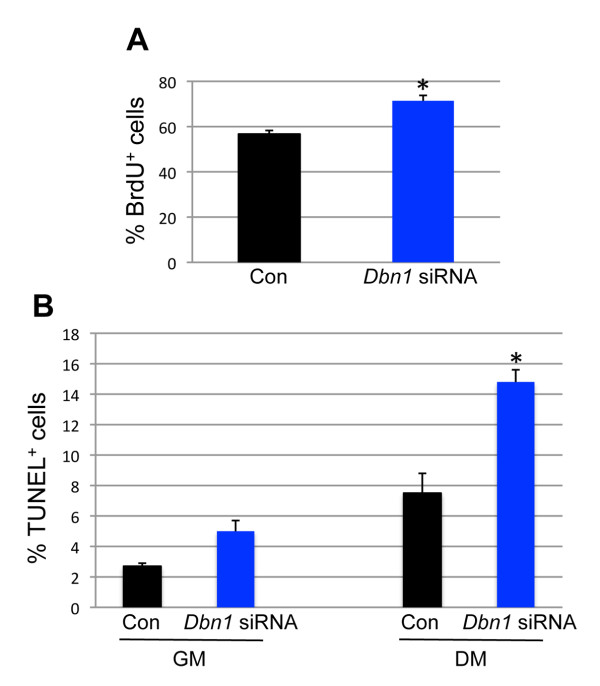
**Cell proliferation and apoptosis in primary myoblasts depleted of drebrin**. **(A) **Primary myoblatsts were transfected with control or *Dbn1*-specific siRNA. Cells were cultured for 36 hours in growth medium (GM), then exposed to bromodeoxyuridine (BrdU) for 2 hours and finally fixed and stained with antibody against BrdU. Values shown are means ± SD of triplicate determinations. **(B) **Primary myoblasts were transfected with control (Con) or *Dbn1*-specific siRNA. Cells were cultured in GM for 36 hours or in differentiation medium for 48 hours. Values shown are means ± SD of triplicate determinations. *P < 0.01 by Student's *t*-test.

The possibility that defective differentiation of myoblasts depleted of drebrin was due to alterations in cell proliferation or apoptosis was examined by measuring BrdU incorporation and by terminal deoxynucleotidyltransferase deoxyuridine triphosphate nick-end labeling (TUNEL) assay, respectively. BrdU incorporation was slightly, but significantly, increased in primary myoblasts in GM treated with anti-*Dbn1 *siRNA relative to control cells (Figure 4A). Approximately 2% of cells in control cultures in GM were TUNEL-positive, and this was increased to about 5% in drebrin-depleted cultures (Figure 4B). Forty-eight hours after switching to DM, about 8% of cells in the control cultures were TUNEL-positive, whereas approximately 15% of cells in drebrin-depleted cultures were undergoing apoptosis (Figure 4B). Although these increases in cell proliferation and apoptosis may contribute to the defective myogenesis seen in cells depleted of drebrin, they are insufficient to explain the quantitatively much stronger effects on myotube formation.

### The drebrin inhibitor BTP2 blocks myoblast differentiation and myotube formation

**Figure 5:**
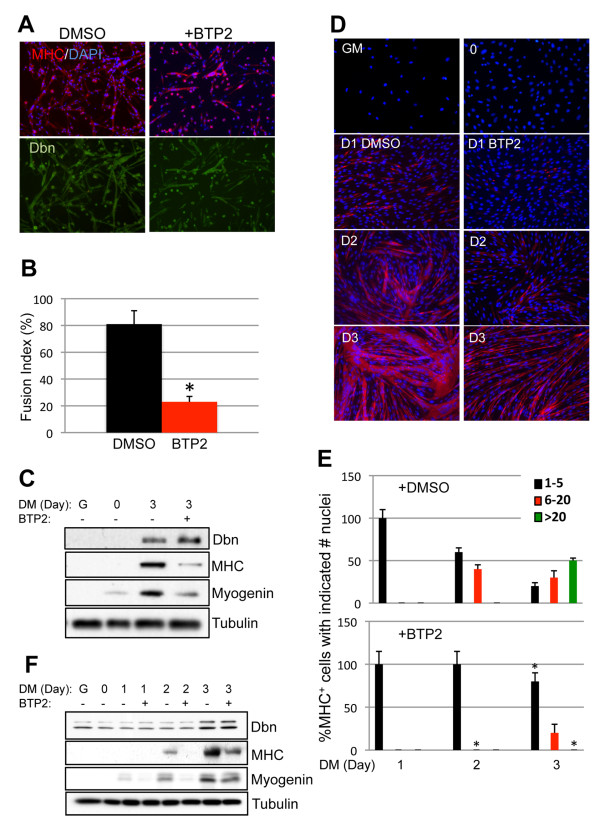
**The drebrin antagonist BTP2 inhibits myoblast differentiation**. **(A) **Primary myoblasts were cultured in differentiation medium for 72 hours in the presence or absence of the drebrin inhibitor BTP2 (5 μM), then fixed and stained for myosin heavy chain (MHC) (red) and drebrin (green). Nuclei were counterstained with 4',6-diamidino-2-phenylindole (blue). **(B) **Quantification of the fusion index in cultures from part **(A)**. Values shown are means ± SD of triplicate determinations. *P < 0.01. **(C) **Cultures parallel to those in part **(A) **were examined by Western blot analysis for drebrin, myogenin and MHC production. Tubulin was used as a loading control. **(D) **through **(F) **are the same as **(A) **through **(C)**, except C2C12 cells were used. The asterisks in **(E) **refer to differences between dimethyl sulfoxide- and BTP2-treated cells in each of the indicated categories.

BTP2 is a 3,5-bis(trifluoromethyl)pyrazole derivative that binds directly to drebrin and blocks actin rearrangements induced by forced expression of drebrin in CHO cells [46,47]. We treated C2C12 cells and primary myoblasts with BTP2 to assess its effects on formation of myotubes and expression of myogenin and MHC. In both primary myoblasts and C2C12 cells, treatment with BTP2 throughout a 3-day differentiation time course resulted in formation of myotubes with much fewer nuclei than those formed by vehicle-treated cells (Figures 5A, B, D and 5E). Expression of myogenin and MHC was also diminished by BTP2 (Figures 5C and 5F). Therefore, treatment of myoblasts with BTP2 produced a phenotype similar to knockdown of drebrin, although BTP2 did not alter drebrin protein levels (Figures 5C and 5F).

**Figure 6:**
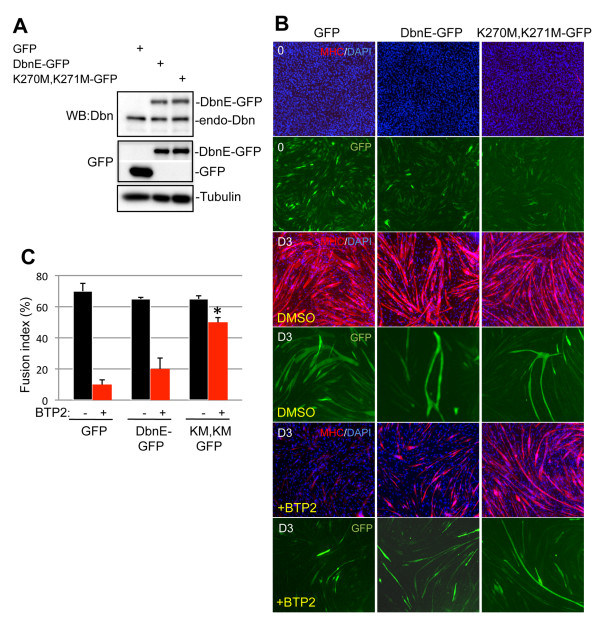
**Expression of a drebrin mutant insensitive to BTP2 rescues myotube formation in BTP2-treated C2C12 cells**. **(A) **C2C12 cells were transfected with vectors encoding GFP, drebrin E-GFP or drebrin E (K270M, K271M)-GFP. Transfection efficiency was measured as > 70% by visualization of GFP. Forty-eight hours after transfection, expression of exogenous and endogenous drebrin was examined by Western blot analysis with antibodies to GFP or drebrin (Dbn). **(B) **Forty-eight hours after transfection, cells were switched to differentiation medium with or without 5 μM BTP2 as indicated. Seventy-two hours later, cultures were fixed and immunostained for myosin heavy chain (MHC; red). GFP and exogenous drebrin expression were visualized by direct fluorescence (green). Nuclei were counterstained with 4',6-diamidino-2-phenylindole (blue). KM, KM-GFP; drebrin E (K270M, K271M)-GFP. **(C) **Quantification of the fusion index in cultures from **(A)**. Values shown are means ± SD of triplicate determinations. *P < 0.01 as compared to BTP2-treated GFP-expressing cultures.

To prove that drebrin was the target of BTP2 that led to inhibition of myogenesis, C2C12 cells were stably transfected with expression vectors encoding either wild-type drebrin E fused to GFP or a mutant form of drebrin E that cannot bind BTP2(K270M, K271M) [46], also fused to GFP. A vector that expressed GFP alone was used as an additional control. Western blot analyses with antibodies to drebrin showed that the levels of drebrin E-GFP and drebrin E(K270M, K271M)-GFP were very similar to each other and to the levels of endogenously expressed drebrin (Figure 6A). As expected, GFP-expressing cells formed myotubes efficiently in a manner that was inhibited by treatment with BTP2 (Figures 6B and 6C). Expression of drebrin E-GFP had only a very modest ability to rescue the inhibitory effects of BTP2 (Figures 6B and 6C), suggesting that the concentration of the drug used was sufficient to inhibit the approximately twofold increase in overall drebrin levels in these cells. In contrast, expression of drebrin E(K270M, K271M)-GFP strongly rescued the fusion index of BTP2-treated cells to near that of untreated cells (Figures 6B and 6C). These results indicate that the major target of BTP2 in the inhibition of myogenesis is drebrin.

**Figure 7:**
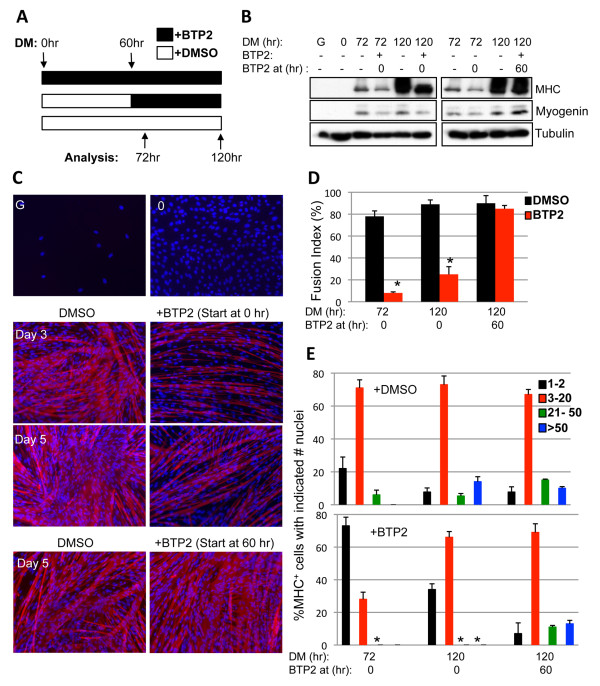
**BTP2 does not block late stages of myoblast differentiation**. **(A) **Experimental scheme outlining the treatment of C2C12 cells with BTP2 and subsequent analysis. **(B) **Western blot analysis of C2C12 cultures in growth medium (G), at the time of transfer to differentiation medium (DM) (time 0) or incubated in DM plus BTP2 from either time 0 or 60 hours and harvested at either 72 or 120 hours. **(C) **Cultures parallel to those in part **(B) **were fixed and stained for myosin heavy chain (MHC; red) and 4',6-diamidino-2-phenylindole (blue). **(D) **Quantification of the total fusion index in cultures from part **(C)**. *P < 0.05 as compared with dimethyl sulfoxide (DMSO) control at the same time point. **(E) **Quantification of myotube formation. Values shown are means ± SD of triplicate determinations. *P < 0.05 as compared to the DMSO control at the same time point in the graph above.

To gain further insight into drebrin function, 5-day (120 hours) differentiation time courses were performed under the following conditions: (1) BTP2 was present in the cultures continuously from the time of switch to DM (designated as time 0), (2) BTP2 was added to cultures after 60 hours in DM without BTP2 and was maintained until the final 120-hour time point or (3) DMSO was present in the cultures as a vehicle control throughout the entire 120-hour time course (see Figure 7A). Cells were harvested at 72 and 120 hours for analysis of myotube formation and production of myogenin and MHC. Relative to DMSO vehicle controls, cells treated with BTP2 throughout the time course (condition 1) displayed a reduced overall fusion index, smaller myotubes with fewer nuclei and lower levels of myogenin and MHC at both 72 and 120 hours (Figures 7B through 7E). The number of nuclei per myotube, and levels of MHC, increased between 72 and 120 hours in DM in both control cultures and cultures continuously treated with BTP2, but the latter cultures differentiated defectively by these measures at both time points, relative to control cells at the same time point (Figures 7B and 7E). In contrast to the effects seen with cells treated continuously with BTP2, cultures in which BTP2 treatment began at 60 hours were indistinguishable from DMSO control cultures when analyzed at 120 hours. Levels of MHC (but not myogenin) increased in control cultures between 72 and 120 hours, but this increase was unaffected when BTP2 was added at the 60-hour time point (Figure 7B). Similarly, the number of nuclei per myotube increased significantly between 72 and 120 hours in control cultures, and myotubes with very large numbers of nuclei formed during this period; however, inclusion of BTP2 at 60 hours had no effect on this process (Figures 7C through 7E). The overall fusion index of control cultures did not increase between 72 and 120 hours, so the increase in nuclei per myotube at 120 hours presumably reflects myotube-myotube fusion. The results shown in Figure 7 reveal that drebrin functions to promote myogenesis relatively early in the differentiation process and that it is dispensable in a later phase characterized by increased levels of MHC and myotube-myotube fusion.

### Drebrin localization

**Figure 8:**
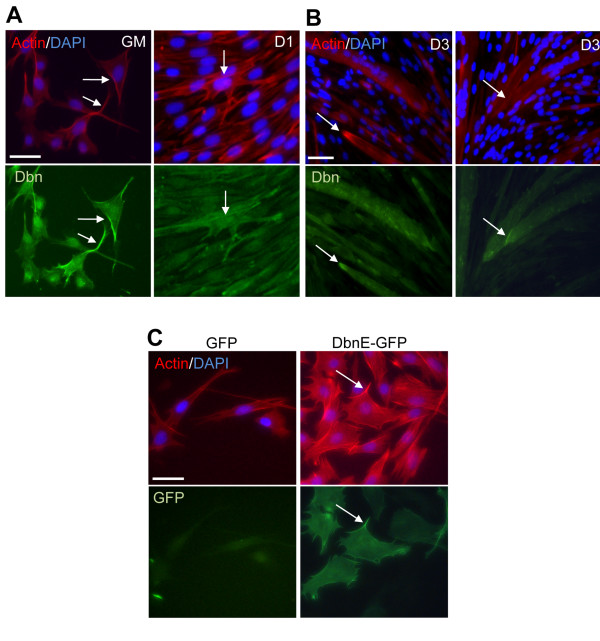
**Drebrin localization in growing and differentiating C2C12 cells**. **(A) **C2C12 cells in growth medium (GM) or differentiation medium (DM) for 1 day (D1) were fixed and immunostained for endogenous drebrin (Dbn1; green) and F-actin (red). Arrows indicate areas of overlapping enrichment for drebrin and F-actin in cell processes (GM) and at sites of cell-cell contact (D1). **(B) **C2C12 cells in DM for 3 days (D3) were fixed and immunostained as in part **(A)**. Arrows show areas of overlapping enrichment for drebrin and F-actin at the tips of myotubes (left panels) and at sites of cell-cell contact between myotubes (right panels). **(C) **C2C12 cells were transfected with vectors encoding GFP or drebrin E-GFP. Cells were fixed, and actin was visualized by staining with phalloidin (red). GFP and exogenous drebrin expression were visualized by direct fluorescence (green). Nuclei were counterstained with 4',6-diamidino-2-phenylindole (blue). Arrows show enrichment for drebrin-GFP and F-actin. The exposure time for cultures in GM was three times as long as it was for cultures in DM (1.14 seconds vs 300 to 400 milliseconds, respectively) to visualize the lower levels of drebrin protein in cells in GM.

We determined drebrin's subcellular localization during differentiation of C2C12 myoblasts by immunofluorescence. Although drebrin levels are relatively low in low-density cultures in GM (Figure 1), drebrin was concentrated in cell projections and at cell cortices, areas that also accumulated F-actin, as revealed by costaining with phalloidin (Figure 8A). A similar result was obtained by direct visualization of drebrin E-GFP transiently expressed in C2C12 cells (Figure 8C). In higher-density cultures in DM for 24 hours (a condition in which such cells are preparing to fuse), drebrin was somewhat diffusely distributed in the cytoplasm but was concentrated at sites of cell-cell contact (Figure 8A). In myotubes after 3 days in DM, drebrin was again concentrated at sites of cell contact between myotubes and also at the tips of myotubes (Figure 8B).

## Discussion

Myoblast differentiation involves expression of the muscle-specific transcriptional program and changes in cellular morphology, most strikingly among the latter, fusion into multinucleated myofibers. The p38 MAPK signaling pathway promotes myogenic differentiation, including myoblast fusion [6,8]. The reported substrates of p38 that are involved in myogenesis are all involved with expression of muscle-specific genes [11-14,16-18], yet few p38 target genes that regulate myogenesis have been identified. In this report, we identify *Dbn1*, encoding drebrin, as a gene that is induced during myoblast differentiation in an SB203580-sensitive manner and that promotes the differentiation process.

The inhibition of *Dbn1 *induction by SB203580 suggests that one or more p38-regulated transcription factors are involved, directly or indirectly, in the expression of *Dbn1 *during myoblast differentiation. MyoD and Mef2 family members are well-established as p38-regulated transcription factors that are central to muscle-specific gene expression and myogenesis, and they are therefore potential candidate regulators of *Dbn1 *expression [8]. Investigators in a recent study identified MEK5/ERK5 as a signaling pathway in myoblasts that induces expression of the transcription factors Klf2 and Klf4. Klf2 and Klf4 in turn regulate a set of target genes involved in myoblast fusion, largely independently of MyoD and Mef2, and *Dbn1 *was among this set of ERK5 → Klf2/Klf4 target genes [48]. Klf4 can be posttranslationally activated by p38 MAPK [49], providing another potential explanation for the sensitivity of *Dbn1 *induction to SB203580. The nonreceptor tyrosine kinase FAK is important for myoblast maturation and fusion, and *Dbn1 *induction failed to occur properly in primary myoblasts in which fusion was inhibited by forced expression of FAT (Focal Adhesion Targeting, a naturally occurring dominant-negative variant of FAK) (see Table S1 in [9]). Taken together, *Dbn1 *expression has been associated with myogenesis in several systems, but its regulation is likely to be complex, integrating input from multiple signaling pathways and, potentially, multiple transcription factors.

RNAi-mediated depletion of drebrin or inhibition of drebrin function with the chemical inhibitor BTP2 led to decreases in expression of myogenin and MHC and inhibition of myotube formation. Drebrin encodes an F-actin side-binding protein and is best known for its role in remodeling actin to promote maturation of filopodia into dendritic spines during synaptogenesis in developing neurons [27,28]. Production and remodeling of F-actin-enriched structures is a key event in myoblast fusion [50], and it is logical to posit that drebrin may participate in such events. However, the fact that depletion or chemical inhibition of drebrin reduced the levels of myogenin, which is itself required for differentiation, raises the possibility that the diminished fusion into myotubes seen upon loss of drebrin function may occur as a consequence of a more generalized block in differentiation due to suboptimal myogenin induction. The decrease in MHC expression seen in drebrin-inhibited cells and the fact that, based on time courses of BTP2 treatment, drebrin function was not required after 60 hours in DM, supports the idea that regulation of myogenin expression is likely involved with at least some portion of drebrin's actions in myogenesis. Consistent with this notion, there is evidence to suggest that proper regulation of cytoskeletal structures may be important in muscle-specific gene expression. For example, muscle-specific gene expression is hindered when myoblasts are treated with the F-actin remodeling inhibitor latrunculin B or depleted of the microtubule-binding protein EB1 [21,24]. Additionally, Gussoni and colleagues [51] reported that C6ORF32, a cytoplasmic protein induced during myoblast differentiation that promotes filopodia formation, is important for expression of myogenin and MHC. Furthermore, depletion in cultured mammalian myoblasts of factors specific for myoblast fusion in *Drosophila *(for example, Dock1, Brag2) not only blocks formation of multinucleated cells but also decreases the percentage of cells that express muscle-specific markers [21,52]. It seems logical that at least some aspects of muscle-specific transcription and myoblast fusion would be coordinately regulated via cytoskeletal dynamics, and, if so, drebrin would be well-placed to participate in such coordinated regulation. Consistent with this likelihood, our findings indicate that drebrin in myoblasts is enriched in cellular projections, cell cortices and regions of cell-cell contact, all sites where we found F-actin was also concentrated.

Drebrin interacts with several other proteins that have been implicated in myoblast differentiation and fusion. For example, drebrin binds the microtubule plus-tip binding protein EB3 in axonal growth cones. This interaction is important for growth cone formation and axonal growth and provides a link between F-actin and dynamic microtubules [30,33]. EB3 is important for myoblast fusion, and its expression is induced during myoblast differentiation with kinetics similar to *Dbn1 *induction [34]. Drebrin also binds to the scaffold protein Homer [35]. Homer2b promotes signaling by the calcineurin/Nuclear Factor of Activated T Cells (NFAT) pathway and enhances myoblast differentiation and myotube formation [36]. NFAT family members regulate several aspects of myogenesis [7]. Interestingly, the drebrin inhibitor BTP2 was originally identified in a screen for small-molecule antagonists of NFAT activity and was subsequently shown to bind directly to drebrin [47]. In addition to their ability to regulate calcium signaling, Homer proteins associate with Cdc42 [35], a small GTPase involved in myoblast differentiation and fusion [37,38]. Finally, drebrin binds to the cytoplasmic tail of the chemokine receptor CXCR4 [40]. CXCR4 promotes myoblast and myocyte migration [39]. Given the plethora of drebrin interactions of potential relevance, extensive structure-function studies are likely necessary to identify the most important ones for drebrin's actions in myogenesis.

## Conclusions

In this study, we identified *Dbn1 *as a gene induced during myoblast differentiation in a p38 MAPK-dependent manner. *Dbn1 *encodes drebrin, an actin-binding protein that localizes to cellular extensions, cell cortices and sites of cell-cell contact. Inhibition of drebrin function, either by RNAi-mediated depletion or by the small-molecule inhibitor BTP2, reveals a role for drebrin in myogenic differentiation.

## Abbreviations

DM: differentiation medium; DMEM: Dulbecco's modified Eagle's medium; GFP: green fluorescent protein; GM: growth medium; MHC: myosin heavy chain; PBS: phosphate-buffered saline; RNAi: RNA interference; RT-PCR: reverse transcriptase polymerase chain reaction; siRNA: small interfering RNA.

## Competing interests

The authors declare that they have no competing interests.

## Authors' contributions

AM, DS and RSK designed the experiments. AM and DS carried out the experiments. AM, DS, WZ and RSK analyzed the data. HY and TS generated and provided critical reagents. AM and RSK wrote the paper. All authors read and approved the final manuscript.
